# The Role of Forestry-Based Policies in Alleviating Relative Poverty in the Rocky Desertification Area in Southwest China

**DOI:** 10.3390/ijerph192316049

**Published:** 2022-11-30

**Authors:** Yifan Wang, He Li, Rong Zhao

**Affiliations:** Research Institute of Forestry Policy and Information, Chinese Academy of Forestry, Beijing 100091, China

**Keywords:** forestry-based poverty alleviation policy, relative poverty, rural households, China

## Abstract

China has put forward a series of forestry-based poverty alleviation policies, aiming to alleviate poverty and improve the livelihoods of rural households, especially in backward and ecologically fragile areas with rich forest resources. Based on field survey data, we used an empirical analysis method to investigate the role of forestry-based policies in alleviating the relative poverty of rural households in the rocky desertification area in southwest China. The Logit regression results demonstrate that forestry-based poverty alleviation policies are significant at alleviating the relative poverty of rural households, and there are differences in the degree and significance of the impact of various forestry-based poverty alleviation policies. In particular, the forestry industry support (FIS) policy, the ecological forest rangers (EFRs) policy, and the Sloping Land Conversion Program (SLCP) can significantly decrease the incidence of relative poverty of rural households, while the effect of the Public Benefit Forest Compensation Program (PBFC) on decreasing the incidence of relative poverty is not significant. The findings contribute to a better understanding of the role and effectiveness of China’s forestry-based poverty alleviation policies, and can provide a reference for optimizing the forestry poverty alleviation policies for the specific area and even the whole nation, as well as provide experience for worldwide poverty alleviation by forestry.

## 1. Introduction

Over the last few decades, poverty has drawn tremendous attention around the world. “No poverty” is listed as the first goal of the sustainable development goals (SDGs) of the UN. As the largest developing country in the world, China has achieved world-renowned success in poverty alleviation, announcing the elimination of absolute poverty at the end of 2020 [1]. After that, China’s efforts to battle poverty switched from ending absolute poverty to alleviating relative poverty [2]. Poverty is a major socio-economic problem, and one cause of poverty is ecosystem degradation [3]. In particular, forests, as an integral part of the Earth’s terrestrial ecosystem, are often linked to regional poverty and livelihoods in a subtle way. A growing number of attempts regarding the implementation of forest-related ecosystem restoration programs have been made to tackle the complex problems of poverty and ecological degradation. In recent years, forests have indeed played a notable role in addressing natural issues as well as socio-economic issues in several countries and regions. Countries have increasingly noted the irreplaceable role of forestry in performing the dual function of poverty reduction and ecological conservation, starting to develop a series of forestry-based policies in response to global challenges such as extreme poverty, climate change, and ecological restoration. China has carried out a series of innovative initiatives in this regard without exception.

The rocky desertification area in southwest China is a typical area with backward economic development and extremely fragile ecology [4], which was the region with the largest number of poor people, the deepest poverty, and the heaviest tasks of ecological restoration in China in the past. Before the end of 2020, forestry has made a great contribution to winning the battle against absolute poverty in the rocky desertification area. In the new poverty alleviation era, the rocky desertification area is still facing the risk of relative poverty due to the constraints of natural resources and the ecological environment. The particularity of alleviating relative poverty in the rocky desertification area is that ecological protection and green development must be the premise, which means that forestry inevitably has more responsibilities in alleviating relative poverty in this area. In recent years, China has implemented several poverty alleviation policies based on forestry [5,6], and these policies are most typical and representative in the rocky desertification area in southwest China.

A great deal of research has focused on the relationship between forests and poverty alleviation [7,8,9]. Forests provide not only food and fuel, but also an important source of income for many rural households in areas rich in forest resources. For example, households with forest-based livelihoods have a higher income than those without forest-based livelihoods in the Himalayan region of rural Pakistan [10]. Forests are widely regarded as an important source of revenue, and there is no doubt that forests are critical to global efforts to alleviate poverty, even though there may be an uneven distribution of benefits from forests. Nevertheless, the extent to which forests can alleviate local poverty, increase income, and improve livelihoods remains controversial. A certain proportion of poor people in rural areas seem to rely on forest resources, suggesting a probable link between forests and poverty [11]. Furthermore, there is controversy about whether forest restoration can improve ecosystem services and enhance livelihoods at the same time [12]. Of course, the poverty alleviation effects of forests do not work at all times and places. Because of geographical, social, economic, and other factors, the role of forests in poverty alleviation varies significantly in different local environments [11,13]. Global forces of change may bring opportunities and risks to forest-reliant households and have impacts on their poverty dynamics [14]. Other research focuses on special factors such as forestry decentralization and forest tenure rights that may have impacts on forest income and poverty alleviation. Forestry decentralization has been widely promoted around the world, although the direct effect of the measure has stirred up controversy [15]. State-owned forests and community-owned forests have clear differences in the generation of forest income [16]. Interventions to strengthen forest property rights have proven to have a positive impact on poverty alleviation or income growth [17]. As a result, a better understanding of contexts and how they shape change in forest ecosystems is essential for the design and implementation of more effective forestry-based policies to alleviate poverty [13].

Countries around the world are exploring forestry-based strategies and policies to alleviate poverty or improve livelihoods. For example, the role and the potential of non-timber forest products (NTFPs) collection in poverty alleviation and livelihood improvement is widely recognized [18,19,20,21,22]. Moreover, community forestry is considered as a common pattern of forest management around the world, aiming to achieve the win–win goals of forest conservation and regional poverty alleviation, especially in developing countries. The implementation of community-based forest management (CBFM) initiatives in several counties has achieved success, which is well-known in Nepal, India, etc. [23]. Meanwhile, China has put forward several poverty alleviation policies or programs related to forests [24], such as the ecological forest rangers (EFRs) policy, the Sloping Land Conversion Program (SLCP), and the Public Benefit Forest Compensation Program (PBFC). Generally, these policies combine ecological and economic objectives and functions [25]. Research indicates that different forestry-related poverty alleviation policies have different impacts on participating rural households [5]. Although there have been some studies on forestry poverty alleviation policies, most of them have only focused on the impact of a single policy or measure on poverty alleviation or livelihood improvement of rural households [6]. Nevertheless, more comprehensive research on the forestry-related poverty alleviation policies in China is still limited.

This paper aims to analyze the impact of China’s forestry-related poverty alleviation policies on alleviating the relative poverty of rural households in a specific poverty-stricken area in China. In this study, four typical counties that acquire pairing-off assistance in poverty alleviation from the National Forestry and Grassland Administration, China, were taken as the study area, and a total of 303 household surveys were conducted in the four counties. Moreover, we expect to further discussed the other factors that may be correlated with the occurrence of relative poverty of rural households. We hope our conclusions could be beneficial to a better understanding of the role and effectiveness of China’s forestry poverty alleviation policies, and provide a valuable reference for optimizing forestry poverty alleviation policies in China, as well as provide experiences for the worldwide forestry-based poverty alleviation practices.

## 2. Materials and Methods

### 2.1. Study Area

This study was conducted in four counties in rocky desertification areas in southwest China. The four counties were Longsheng county and Luocheng county in Guangxi Province, and Dushan county and Libo county in Guizhou Province, which were poverty-stricken counties acquiring paired-up assistance in poverty alleviation from the National Forestry and Grassland Administration, China. Thus, the implementation of forestry poverty alleviation policies in this area is typical and exemplary from a national point of view. The basic information on the four counties is shown in Table 1.

The rocky desertification area in southwest China has a particularity in natural conditions, resource endowment, and social-economic environments, especially having difficulty in controlling rocky desertification [26,27]. Forestry ecological poverty alleviation actions in this area are explored in accordance with local conditions, forming multi-level and multi-dimensional forestry ecological poverty alleviation patterns with regional characteristics. In the past few years, China has put forward a series of forestry poverty alleviation policies, intending to explore paths to increase forestry income on the premise of protecting forest resources, rather than using timber harvest as a pathway. In this study, we focused on four forestry poverty alleviation policies that have been widely implemented in the rocky desertification area.

### 2.2. Typical Forestry-Based Poverty Alleviation Policies in China

There are several typical forestry-based poverty alleviation policies in China, and of course they are widely implemented in the rocky desertification area.

- Policy1: The forestry industry support policy

The forestry industry support (FIS) policy is that the forestry industry development funds from the government are dedicated to supporting the development of the forestry industry in poor areas, and prioritize the participation of poor households in forestry industry construction in the form of subsidies to encourage the development of forestry planting, processing, etc.

- Policy2: The ecological forest rangers policy

The ecological forest rangers (EFRs) policy is that the government uses special transfer payments to purchase labor services within the scope of the impoverished population with poverty registration in poverty-stricken counties to employ poor populations to participate in forest protection [28].

- Policy3: The Sloping Land Conversion Program

The Sloping Land Conversion Program (SLCP) is a policy that aims to motivate farmers to return farmland into forestland through cash subsidies, which can improve the ecological environment, adjust the structure of rural land use, and increase rural household income at the same time [6].

- Policy4: The Public Benefit Forest Compensation Program

The Public Benefit Forest Compensation Program (PBFC) is a policy in which the central and local governments provide compensation for rural households according to the area of the public benefit forests under the protection of rural households, which is in the form of financial special funds.

### 2.3. Data Sources

The data used in this paper come from the rural household surveys conducted by the research team in the four counties. A total of 320 household samples were derived from 32 villages of 16 townships in the four counties in early 2021 by random sampling. After eliminating invalid samples, the final valid sample size was 303, and the effective rate of the samples was 94.69%. Information on household characteristics, land resource endowment, and household income were involved in the survey. The sample distribution proportion of the four counties was between 23.43% and 26.73% (Table 2).

### 2.4. Variable Setting

This study used the 0–1 binary variable “whether to fall into relative poverty” measured by income dimension as the dependent variable of the model (Table 3). If the per capita income of the rural household was lower than the relative poverty standard, it was regarded as relative poverty, and the value was assigned as 1, otherwise, it was assigned as 0.

In terms of the relative poverty line, different countries and economic entities set their own relative poverty line based on their socioeconomic development levels. Ordinally, the more developed countries or economic entities set the relative poverty line with a higher proportion of income [29]. The income-ratio method has been a mainstream method of identifying relative poverty in more developed countries, regions, and economic entities such as the European Union and OECD, taking 50% of the median household income of the whole population as the relative poverty line [30]. In this study, because of the existence of individual extremely high values of household income, the income was not characterized as normally distributed. In other words, 50% of the median and 50% of the average value of the per capita household income had a visible gap, the former about CNY 5004 and the latter about CNY 5906. To ensure that the setting of the relative poverty line contained more information, namely the larger relative poverty group, the higher relative poverty line may be the better setting. Then, 50% of the average household income by household size, rather than 50% of the median per capita household income, was set as the relative poverty line in this study. To examine how the relative poverty rate changes and how the effects of forestry-based poverty alleviation policies change under the higher relative poverty line, the lines of 50%, 60%, and 70% standard were set, under which the ratios of relatively poor rural households were 20.8%, 27.7%, and 35%, respectively (Table 4).

This study mainly investigates the impacts of forestry poverty alleviation policies on the relative poverty of rural households. Four binary independent variables in terms of forestry poverty alleviation policies were selected, including “whether to achieve forestry industry support”, “whether the family member has been an ecological forest ranger”, “whether to participate in the Sloping Land Conversion Program (SLCP)” and “whether to participate in the Public Benefit Forest Compensation Program (PBFC)” (Table 3). Specifically, among the four specific forestry-related poverty alleviation policies, about 22.44% of rural households achieved forestry industry support in the past two years, about 75.58% had family members holding ecological forest ranger positions, about 13.53% participated in the SLCP, and about 24.42% participated in the PBFC, shown as the diagonal elements in Table 5. Moreover, the percentage of rural households participating in the combination of double policies is shown as the off-diagonal elements contained in Table 5.

The control variables of household characteristics, land resource endowment, and other relevant variables were involved. In particular, the variables of household characteristics included “age of household head”, “education level of household head”, “number of on-farm employment”, and “household labor force ratio”. The variables of land resource endowment characteristics included “cultivated land area” and “forestland area”. In addition, “proportion of forestry income in household income” was added as a control variable to investigate the correlation between the proportion of forestry income in household income and the relative poverty of rural households (Table 3).

Among the control variables, the average age of the household head ranged from 51 to 52 years old, and the average education level ranged between primary and junior high school levels. Regarding the characteristics of the sample rural households, the average household size amounted to 4.261. The average number of laborers in the household was 2.574, of which the off-farm and on-farm were 1.254 and 1.32, respectively, and the average proportion of the number of laborers in the household to the household size was 63.6%. In terms of land resource endowment, the average area of cultivated land owned by the rural households was 0.379 ha, with the largest household owning 2.933 ha, and the average area of forestland owned by rural households was higher than the area of cultivated land, reaching 0.857 ha, with the largest household owning 14.867 ha (Table 4).

### 2.5. Research Methodology

The Logit model, also known as logistic regression, is one of the discrete choice models, which is normally applied to predict the probability of whether the event occurs or not [31]. In this study, the Logit model was used to analyze the impact of forestry-based policies on alleviating the relative poverty of rural households. In other words, we investigated whether forestry-based policies have significantly different impacts on policy participants and non-participants in the occurrence of relative poverty. The modeling process, as well as the data description and analysis, were done using the Stata 16.0 software (StataCorp LLC, College Station, TX, USA).

The independent variable Y represents whether the sample rural household is falling into relative poverty. The value of this independent variable is 0 or 1. When the rural household is falling into relative poverty, the value of Y is 1, and vice versa, the value of Y is 0. Therefore, the probability that the rural household is falling into relative poverty is as follows (Equation (1)).

(1) $$Prob\left(Y\right)=\frac{e^{\beta_{0}+\beta_{1}X_{1}+\beta_{2}X_{2}+\ldots \beta_{k}X_{k}}}{1+e^{\beta_{0}+\beta_{1}X_{1}+\beta_{2}X_{2}+\ldots \beta_{k}X_{k}}}$$

Through transformation, the numerator in the right hand of Equation (1) can be represented as a form of probability that the rural household is falling into relative poverty to that is not falling into relative poverty (Equation (2)).

(2) $$e^{\beta_{0}+\beta_{1}X_{1}+\beta_{2}X_{2}+\ldots \beta_{k}X_{k}}=\frac{P}{1-P}$$

By taking both sides of Equation (2) logarithmically at the same time, Equation (3) is obtained, which is the ultimate form of the Logit regression model. $X_{1}$, $X_{2}$, …, $X_{k}$ are the explanatory variables, $\beta_{0}$, $\beta_{1}$, $\beta_{2}$, …, $\beta_{k}$ are the parameters to be estimated, and ε is the random disturbance term (Equation (3)).

(3) $$ln\left(\frac{P}{1-P}\right)=\beta_{0}+\beta_{1}X_{1}+\beta_{2}X_{2}+\ldots +\beta_{k}X_{k}+\varepsilon$$

## 3. Results

### 3.1. Household Forestry Income

In general, the source of household income can be classified into four types, namely production income, wage income, property income, and transfer income. Similarly, the classification criterion is applicable to forestry income. Forestry production income mainly comes from timber forest, forest fruit, planting and breeding under the forest, etc. Forestry wage income is mainly derived from the labor subsidy of forestry-related public welfare posts such as ecological forest rangers. Forestry property income normally comes from the dividend in the form of forestland transferred to cooperatives and the rental of forestland. Forestry transfer income mainly comes from forestry-related compensation or subsidies from the government departments such as the Sloping Land Conversion Program (SLCP) and the Public Benefit Forest Compensation Program (PBFC).

Overall, the average annual forestry income of 303 households is CNY 9873.99, accounting for 27.8% of the average annual income of the households. In particular, forestry wage income accounts for the majority of forestry income, which reaches CNY 7013.99 and accounts for 71.04%, and is mainly derived from subsidies for ecological forest rangers. The target of the ecological forest rangers (EFRs) policy is the former deeply poor rural households; therefore, it covers a wide range of local poor populations, with about 75.58% of households having one family member as the EFR. Meanwhile, the average forestry production income in the sample is CNY 2530.43, accounting for 25.63% of the total income. Forestry industry development still makes a limited contribution to the growth of forestry income, as a consequence of its long production cycle and failure to benefit most poverty-stricken households. As for forestry production income, it should be noted that as the household income in 2020 is measured in this sample, and the logging cycle of a timber forest is generally long, many households did not harvest timber in 2020, so there is no relevant income included in the forestry productive income of that year. However, the potential income of timber forest in the future, as a part of household income, is not included in the income of the current year, which is an underestimate of the ability of households to obtain a forestry production income to a certain extent. Additionally, the average forestry transfer income and forestry property income are CNY 301.5 and CNY 28.07, respectively, accounting for merely 3.05% and 0.28%, respectively (Table 6).

Furthermore, by comparing the household forestry income of the samples in four counties (Table 7), it is obvious that Dushan county has the highest average household forestry income, reaching CNY 11,930.53, while Libo county and Longsheng county rank after Dushan county and are all above CNY 10,000. The lowest is only CNY 5425.13 in Luocheng county. This situation is almost consistent with the overall economic development levels of the four counties. Specifically, for each type of forestry income, forestry wage income accounted for the largest proportion of forestry income in each of the four counties, all of which are over 60%. Then, the proportion of forestry production income to forestry income is second, only to forestry wage income, in all four counties, but there are differences among counties; for example, the proportion of forestry production income to total forestry income in Luocheng county is 10.66%, while the proportion of that in Longsheng county reaches 35.45%. In addition, the proportion of forestry property income and forestry transfer income in forestry income of the four counties are relatively low; the highest proportion of forestry property income and forestry transfer income is only 0.51% and 3.73%, respectively.

In comparison, it can be found that the forestry income of rural households in the four counties has little difference in forestry property income and forestry transfer income, with both of them accounting for a quite small percentage. The distinction in forestry income is mainly reflected in forestry production income and forestry wage income. Longsheng, Dushan, and Libo counties, where the level of forestry income is relatively high, have a significantly higher absolute value and proportion of forestry production income compared with Luocheng county, while the proportion of forestry wage income of rural households in these three counties is lower than that of Luocheng county, although it is higher in absolute value.

### 3.2. Impacts of Forestry Poverty Alleviation Policies

The Logit regression model is used to investigate whether the forestry poverty alleviation policies and other relevant factors have significant impacts on alleviating the relative poverty of rural households. The Logit regression result under the 50% relative poverty standard is shown in Table 8, including regression coefficients and marginal effects at the means of all of the explanatory variables. The *p* values of the Logit model equal 0, indicating that the model is significant on the whole. As calculated by Stata 16.0 software, the probability of accurate prediction of the model Logit model is 83.83%, implying that the Logit model has a good predictive ability.

In particular, three forestry poverty alleviation policies are significantly correlated with the relative poverty of rural households. Firstly, achieving forestry industry support (FIS) can significantly decrease the probability of falling into relative poverty, of which the marginal effect is −0.107, indicating that the participants in FIS policy are 10.7% less likely to fall into relative poverty than non-participants. A reasonable explanation is that the FIS policy makes participating rural households more deeply involved in the forestry industry development, and they then benefit from forestry production and management. Secondly, “whether the family member has been an ecological forest ranger” also has a significant correlation with the relative poverty of rural households. The government subsidies to EFRs are an extraordinary income for poor rural households in the short term, which may greatly improve their income levels or livelihoods. As a result, the probability of rural households with EFRs falling into relative poverty is significantly decreased by 11.3%. Thirdly, the Sloping Land Conversion Program (SLCP) has a significant impact on relative poverty at a 5% significance level. The marginal effect of −0.152 means that participation in SLCP reduces the probability of falling into relative poverty by 15.2% compared with those non-participants in the SLCP. This is because returning farmland to forestland enables rural households to continue to carry out forestry production activities on the forestland or to engage in other non-agroforestry activities so as to obtain more income after receiving subsidies. However, the Public Benefit Forest Compensation Program (PBFC) has no significant impact on the relative poverty of rural households, as the compensation from PBFC is too weak to make any substantial contribution to the income growth of rural households, and this form of direct compensation does not change their ways of production and lifestyles, or even their income levels.

### 3.3. Impacts of Other Factors

In terms of control variables, there are five control variables that significantly influence the relative poverty of rural households, including the age of the head of the household, the cultivated land area, labor ratio, number of on-farm employees, and proportion of forestry income to total household income (Table 8).

Firstly, the age of the head of the household has a significantly negative impact on the occurrence of relative poverty at the level of 5%, namely the older the head of the household, the lower the probability of falling into relative poverty. For each year of age increase, the probability of relative poverty decreases by 0.4%.

Secondly, cultivated land area negatively affects the relative poverty of rural households at a 1% significance level, and rural households with a larger cultivated land area can obtain more subsistence agricultural products and agricultural income through large-scale farming operations, and thus are less likely to fall into relative poverty.

Thirdly, the household labor force ratio has a significant negative impact on the relative poverty of rural households at a significance level of 1%. The labor force is the fundamental source of the household income. A higher proportion of family labor implies that more people in the households can earn an income. Therefore, the higher the proportion of the labor, the more the per capita income for a rural household, which results in a lower probability of falling into relative poverty.

Fourthly, there is a positive correlation between the number of on-farm employment and relative poverty, which is significant at a level of 1%, which means the more on-farm employment in households, the easier it is to fall into relative poverty. On average, the probability of relative poverty increases by 6.6% for each additional on-farm employment in the household. Thus, it can be inferred that the income level for those who engaged in agroforestry production is much lower than that of off-farm workers. Consequently, more on-farm employment in households may lead to a lower level of per capita income for one rural household and thus make it easier to fall into relative poverty.

In addition, the proportion of forestry income to household income is added as a control variable in the logit regression model. The results show that its coefficient and marginal effect are significantly positive, indicating that the larger the proportion of forestry income, the more likely rural households are to fall into relative poverty. To a certain extent, this reflects that the excessive dependence on forest resources is not conducive to poverty reduction and income growth.

Other control variables, such as the education level of household head and forestland area, do not have significant effects on the relative poverty of rural households. There is no significant correlation between the education level of the head of the household and whether the household is more likely to fall into relative poverty. Thus, the direct effect of forest land area on whether the household is more likely to fall into relative poverty is not significant because the type of forest land is diverse and only economic forestland can generate income.

### 3.4. Robustness Analysis

The differences in the sample distribution of the dependent variable under different relative poverty standards are considered, which in turn may affect the coefficient and the significance of the regression analysis results. For this reason, the robustness of the results of the Logit model under different relative poverty standards is tested by conducting further Logit model regression using 60% and 70% of the average of per capita household income as the relative poverty standard, respectively. As is shown in Table 9, the coefficients and relevant statistics of each variable under 60% and 70% relative poverty standards are reported, indicating that the model is still significant on the whole under the higher relative poverty standard. The negative correlation of the three policies of FIS, EFRs, and SLCP with the relative poverty of rural households are still significant, but there are acceptable distinctions in the coefficients and significance levels under different relative poverty standards. As for the control variables, four variables, namely, the age of the head of the household, the household labor force ratio, number of on-farm employment, and proportion of forestry income to household income, still have significant effects on the relative poverty of rural households, and the only change is that the negative effect of the cultivated land area on the relative poverty of rural households is no longer significant under 60% and 70% relative poverty standards. In summary, the results of the logit model regression analysis have good robustness under the different relative poverty standards.

## 4. Discussion

This study confirms that several forestry poverty alleviation policies in China have a positive effect on alleviating relative poverty or increasing income, which is consistent with some other related research conclusions. For instance, a single policy SLCP was demonstrated to have a significant effect on increasing the income in relevant research [6]. Wu Le and Jin Leshan (2020) assessed the poverty alleviation effects of three forestry-related programs, each corresponding to the SLCP, PBFC, and EFRs policy in this study, on rural households at different income levels by using survey data in three counties in Guizhou Province, China [5]. They found that the SLCP and EFRs policy both have significant increased income effects on rural households, while the role of PBFC in poverty alleviation is shown to be nonsignificant. In particular, the effect of SLCP and EFRs are more significant on rural households at a middle-higher income level and low-income level, respectively. Compared with their research, we examined the effects of four typical forestry-based policies on alleviating relative poverty in a specific area in China, additionally considering the policy of forestry industry support, which in the long term would be a more effective measure for poor rural populations to keep becoming richer through their own efforts. Of course, it is worth discussing the extent to which the poor rural populations could share the interests of the forestry industry development.

Meanwhile, we found a significant correlation between the dependence on forests and low per capita income or the occurrence of relative poverty. On the contrary, the employment opportunities provided by non-agroforestry sectors tended to bring greater income growth to rural households, as a consequence of the low probability of relative poverty occurrence. Relatively poor rural households are more dependent on forests due to a lack of other resources, while higher-income rural households have comparative advantages for off-farm employment [32]. Therefore, it is required that the real effect of forestry-related poverty alleviation policies and the direction of policy adjustment is reconsidered in the future. Forestry industry development is a long-term path for relatively poor rural households to sustainably increase their income. The policy of forestry industry support in the area has greatly stimulated rural households to participate in the planting, processing, and other production stages of forestry, such as the planting of Camellia Oleifera, under-forest medicinal materials, and florals. However, as it realizes a high productivity in forest products, these forest products are faced with the problem of stagnant sales. Although the local government has made great efforts in the sale of forest products, it is still not enough to make forest-reliant rural households eliminate the issue of unsalable forest products. As we know, many factors affect forestry development. In particular, the effects of the market, road, and forest are interdependent and support each other, thus forest conservation and poverty alleviation policies should be coordinated with infrastructure construction to expand the expected forest benefits [33,34].

Obviously, there are limitations in this paper that need to be further improved. First of all, the limited selection of control variables and possible omissions makes the model setting somewhat defective. Considering the small sample size, only the empirical analysis of the overall situation of the sample rural households in the four counties was conducted. Meanwhile, the research data in this paper are cross-sectional data for one year, so the poverty dynamics of those sample rural households cannot be accurately estimated. In future empirical research, we should use panel data and construct multidimensional relative poverty indicators to measure the multidimensional relative poverty index of each rural household and grasp its dynamic change process. Then, it will be feasible to analyze the dynamic impacts of different forestry policies on the relative poverty of rural households by constructing econometric models, in order to further explore more feasible paths for forestry to alleviate relative poverty.

As we know, relative poverty is a long-standing problem in the process of socioeconomic development, and the solution to relative poverty requires more exploration and practice. Forests, as huge ecological assets, play different roles at different stages of socioeconomic development. With the promotion of the ecological civilization construction and rural revitalization strategy in China, forestry poverty alleviation policies might reach a new height, requiring forestry to play a greater role in achieving poverty alleviation and improving livelihoods. Therefore, how to further stimulate the important role of forestry in alleviating relative poverty in rocky desertification areas and how to further realize the ecological value of forests still needs to be thoroughly discussed and studied. After years of investment and assistance in forestry poverty alleviation, both local infrastructure conditions and ideological awareness of relatively poor farmers have changed dramatically in the rocky desertification areas, resulting in a solid foundation for transformation. Therefore, future forestry policies to alleviate relative poverty should pay more attention to stimulating the endogenous development of relatively poor rural households.

This article confirms that forestry policies do play some role in alleviating relative poverty in the specific area, while implying some potential problems that are unfavorable to sustainable income growth. We have pointed out a series of problems in the process of the implementation of forestry poverty alleviation policies in the rocky desertification area, including poor basic support conditions and low diversification of forestry industry, immature linkage mechanism between the participating subjects of forestry industry, inadequate incentive and supervision for ecological forest rangers, and a weak income growth effect of forestry ecological compensation. Therefore, we propose suggestions for optimizing the forestry poverty alleviation policies from four aspects: (1) Strengthening the development dynamics of forestry industry, developing the mechanism of forest resources development and utilization, and innovating the diversified utilization and development patterns of forest resources. (2) Optimizing the linkage mechanism among the participating subjects of the forestry industry, of which the key is to improve the interest linkage mechanism among the participants and to strengthen the mechanism of the forestry industry to benefit poor rural households, and to deepen the forestry science and technology support. (3) Optimizing the incentive of EFRs job compensation distribution and optimizing the supervision and evaluation of EFRs. (4) Improving the forestry ecological compensation mechanism, not only to build and deepen the vertical forestry ecological compensation mechanism, but also to develop the forestry horizontal ecological compensation mechanism.

## 5. Conclusions

The goal of this study is to examine the roles that forestry-based policies in China have played in alleviating relative poverty or improving the livelihoods of rural households. Over the past few years, China has put forward a series of poverty alleviation policies related to forestry, aiming to alleviate poverty and improve livelihoods in backward and ecologically fragile areas with rich forest resources. These forestry poverty alleviation policies that have achieved certain success in southwest China can provide some experiences for the whole country and the world.

Based on the field survey data in four poverty-stricken counties in the rocky desertification area in southwest China, this study uses the Logit regression model to analyze the impact of forestry poverty alleviation policies on alleviating the relative poverty of rural households in the specific area. The empirical analysis results imply that the forestry-based poverty alleviation policies play a significant role in alleviating the relative poverty of rural households on the whole. In particular, three out of four forestry poverty alleviation policies show a significant correlation with a lower incidence of relative poverty for rural households. Under the 50% relative poverty standard, these three policies of the FIS, EFRs, and SLCP can reduce the relative poverty incidence of rural households by 10.7%, 11.3%, and 15.2%, respectively. SLCP plays the greatest role in alleviating poverty, followed by the EFRs policy and the FIS policy. However, the effect of PBFC on decreasing the occurrence probability of relative poverty is not significant. In addition to the main explanatory variables in terms of forestry-based poverty alleviation policies, three control variables, namely the age of the head of the household, cultivated land area, and household labor force ratio, have significantly negative impacts on the incidence of relative poverty, while two control variables, namely on-farm employment and proportion of forestry income to household income, have significantly positive impacts on the incidence of relative poverty.

## Figures and Tables

**Table 1:** Basic information of the four counties (2020).

Indicators	Luocheng	Longsheng	Dushan	Libo
County area (km^2^)	2651	2538	2442.2	2431.8
Population (ten thousand people)	27.27	13.95	26.43	15.49
GDP (one hundred million)	58.35	60.40	129.39	67.94
Forest coverage rate (%)	70.28	81.76	63.5	71.04

**Table 2:** Source and distribution of the sample data.

Province	County	Number	Percentage
Guangxi	Luocheng	78	25.74%
	Longsheng	71	23.43%
Guizhou	Dushan	73	24.09%
	Libo	81	26.73%

**Table 3:** The definition and description of variables.

Variable	Definition	Description
Dependent variable		
Poverty	Whether to fall into relative poverty	0 = No; 1 = Yes
Independent variable		
Policy1	Whether to achieve forestry industry support	0 = No; 1 = Yes
Policy2	Whether the family member has been an ecological forest ranger (EFR)	0 = No; 1 = Yes
Policy3	Whether to participate in the Sloping Land Conversion Program (SLCP)	0 = No; 1 = Yes
Policy4	Whether to participate in the Public Benefit Forest Compensation Program (PBFC)	0 = No; 1 = Yes
Control variable		
Age	Age of household head	Continuous variable
Edu	Education level of household head	1 = Illiterate; 2 = Primary school; 3 = Junior high school; 4 = High school; 5 = College or above
Size	Family size	Positive integer
On_Farm	Number of on-farm employment	Positive integer
LaborRatio	The household labor force ratio	Continuous variable [0, 1]
CultivatedLand	Cultivated land area	Continuous variable
ForestLand	Forestland area	Continuous variable
Pro_forestincome	The proportion of forestry income in household income	Continuous variable [0, 1]

**Table 4:** Basic statistical indexes of variables.

Variables	Mean	S.D.	Min	Max
Poverty				
—Poverty_50	0.208	0.406	0	1
—Poverty_60	0.277	0.448	0	1
—Poverty_70	0.350	0.478	0	1
Policy1	0.224	0.418	0	1
Policy2	0.756	0.43	0	1
Policy3	0.135	0.343	0	1
Policy4	0.244	0.430	0	1
Age	51.14	8.847	25	90
Edu	2.515	0.635	1	4
Size	4.261	1.461	1	8
Labor	2.574	1.086	0	6
Off_Farm	1.254	1.038	0	5
On_Farm	1.320	0.776	0	3
LaborRatio	0.636	0.247	0	1
CultivatedLand (ha)	0.379	0.384	0	2.933
ForestLand (ha)	0.857	1.874	0	14.867
Pro_forestincome	0.278	0.252	0	1

**Table 5:** Percentage of the rural households participating in each policy and the combination of policies.

Policy (%)	Policy1	Policy2	Policy3	Policy4
Policy1	22.44	-	-	-
Policy2	19.14	75.58	-	-
Policy3	3.3	11.55	13.53	-
Policy4	7.92	19.47	7.92	24.42

**Table 6:** The overall situation of all types of forestry income of the sample rural households.

Types of Forestry Income	Mean (CNY)	Proportion (%)
Forestry production income	2530.43	25.63%
Forestry wage income	7013.99	71.04%
Forestry property income	28.07	0.28%
Forestry transfer income	301.50	3.05%
Summation	9873.99	100%

Note: CNY 7.22 ≈ EUR 1 (December 2021).

**Table 7:** Mean and proportion of all types of forestry income for rural households in each county.

Types of Forestry Income	Luocheng County		Longsheng County		Dushan County		Libo County	
Forestry production income	578.21	10.66%	3777.47	35.45%	3141.1	26.33%	2766.91	23.81%
Forestry wage income	4648.72	85.69%	6505.72	61.06%	8293.15	69.51%	8584.36	73.88%
Forestry property income	0	0.00%	0	0.00%	50.68	0.42%	59.32	0.51%
Forestry transfer income	198.21	3.65%	372.18	3.49%	445.6	3.73%	209.14	1.80%
Summation	5425.13	100.0%	10,655.37	100.0%	11,930.53	100.0%	11,619.73	100.0%

**Table 8:** The Logit regression result under the 50% relative poverty standard.

Variables	Logit Model	
	Coefficient	Marginal Effect
Age	−0.040 ** (−1.984)	−0.004 **
Edu	−0.104 (−0.353)	−0.010
CultivatedLand	−0.171 *** (−2.893)	−0.017 ***
ForestLand	−0.005 (−0.751)	−0.001
LaborRatio	−3.539 *** (−4.326)	−0.352 ***
On_Farm	0.667 *** (2.651)	0.066 ***
Pro_forestincome	4.798 *** (5.872)	0.477 ***
Policy1	−1.081 ** (−2.065)	−0.107 **
Policy2	−1.133 ** (−2.350)	−0.113 **
Policy3	−1.527 ** (−2.327)	−0.152 **
Policy4	0.220 (0.508)	−0.022
Constant	2.545 * (1.819)	
Observations	303	
Log likelihood	−109.599	
LR chi2	90.58	
Prob > chi2	0.000	
Pseudo R-squared	0.292	

Note: z-statistics are in parentheses. *** *p* < 0.01. ** *p* < 0.05. * *p* < 0.10. *, **, *** represent the significance levels at 10%, 5%, and 1%, respectively.

**Table 9:** The Logit regression results under the 60% and 70% relative poverty standard.

Variables	60% Relative Poverty Standard	70% Relative Poverty Standard
Age	−0.048 *** (−2.579)	−0.049 *** (−2.730)
Edu	−0.044 (−0.161)	0.007 (0.026)
CultivatedLand	−0.045 (−1.309)	−0.046 (−1.442)
ForestLand	−0.008 (−1.084)	−0.013 * (−1.686)
LaborRatio	−4.043 *** (−5.085)	−3.652 *** (−4.916)
On_Farm	0.873 *** (3.630)	0.807 *** (3.561)
Pro_forestincome	5.528 *** (6.546)	5.868 *** (6.835)
Policy1	−0.759 * (−1.727)	−0.803 ** (−2.001)
Policy2	−1.430 *** (−3.190)	−1.150 *** (−2.750)
Policy3	−0.930 * (−1.780)	−1.069 ** (−2.150)
Policy4	−0.009 (−0.022)	−0.043 (−0.112)
Constant	2.758 ** (2.130)	2.858 ** (2.277)
Log likelihood	−124.157	−135.193
Pseudo R-squared	0.306	0.311
LR statistic	109.42	121.91
Prob (LR statistic)	0.000	0.000

Note: z-statistics are in parentheses. *** *p* < 0.01. ** *p* < 0.05. * *p* < 0.10. *, **, *** represent the significance levels at 10%, 5%, and 1%, respectively.

## Data Availability

The data that support the findings of this study are available upon request from the lead authors. None of the data are publicly available because they contain information that could compromise the privacy of the research participants.
